# From Science to Pleasure: Justifications of the Use of Classic Psychedelics in Finland

**DOI:** 10.1177/14550725251408251

**Published:** 2025-12-30

**Authors:** Mika Tsupari, Aleksi Hupli

**Affiliations:** 1 Sociology, 3835 Helsingin yliopisto, Helsinki, Finland; 2 Faculty of Social Sciences, Emerging Technologies Lab, Tampere University, Tampere, Finland

**Keywords:** drug research, justification analysis, Finland, psychedelics

## Abstract

**Aim:**

This study explores the justification for psychedelics use among 40 Finnish interviewees.

**Methods:**

We conducted and analyzed 40 thematic interviews with Finnish psychedelics users employing justification analysis as an analytical framework. The study investigates justifications as expressed by the users.

**Results:**

We found almost the full spectrum of Boltanski and Thévenot's justification logics represented in the interviewees’ use justifications, with the exception of the logics of the market world. We formed an additional ‘world of self’ to capture justifications connected to various personal experiences, including pleasure, which is often overlooked in clinical research. The participants tended to make a distinction between the use of psychedelics and other drugs or intoxicating substances. The justifications employed ranged from personal level to societal level, and from spiritual to technological justifications. Overall, the justifications appeared largely influenced by the new wave of psychedelic research, self-experienced benefits and ideals of individualism. Reported positive effects included feelings of euphoria and other positive feelings, even when the use was motivated by self-development or self-medication.

**Conclusions:**

The positive and more recreational aspects of psychedelics use are important for users and could be an essential part of the therapeutic benefits of psychedelics, which is often overlooked in the research.

## Introduction

Research on psychedelics and their use is on the rise in the Nordics and worldwide (UNODC 2024). Recent years have seen a substantial increase in knowledge about the consequences of psychedelic use (Johnstad 2021). With the new wave of psychedelic research, we now have a number of studies indicating that psychedelics show potential as a treatment modality for various psychiatric disorders (Nutt & Carhart-Harris 2021; Kajanoja, Kantonen & Kähönen 2024), offering beneficial use values (Tsupari, Hellman & Tammi 2025) and attributions of meaning (Griffiths et al. 2006), as well as a reduction of alcohol use or cessation of smoking (Garcia-Romeu et al., 2019, 2020; Yaden & Griffiths 2021). Their use has not been connected to worsening mental health in population studies (Krebs & Johansen 2013). In the Nordic context, psychedelics are classified as illicit drugs by law in Finland, Sweden, Norway, Denmark and Iceland. Despite this, their potential in therapeutic contexts is researched in Finland and Denmark for alcohol dependency (Yle 2022; EU Clinical Trials Register), in Sweden for palliative care (Uppsala university 2024) and treatment-resistant depression and numerous pre-clinical studies across the Nordic countries (Elsilä et al. 2022; Moliner et al. 2023; Kiilerich et al. 2023). Norway was also part of a multisite clinical trial investigating MDMA (i.e. methylenedioxymethamphetamine)-assisted therapy for post-traumatic stress disorder sponsored by the Multidisciplinary Association for Psychedelic Studies (MAPS).

The new wave of psychedelic research has focused on classical psychedelics, which refer to compounds which cause altered states of consciousness, hallucinations and visions, strong emotional states, the awakening of memories, and mystical experiences (Doblin, 1991). These substances include LSD (i.e. lysergic acid diethylamide), various psilocybin mushrooms, mescaline, DMT (i.e. dimethyltryptamine) and mebufotenin (i.e. 5-MeO-DMT) (Johnson et al., 2019). The most recent European drug report (EUDA 2025) shows that in Denmark 2,8% of the 15–64-year-old population and in Norway 2,3% had used LSD during their lifetime (data based on 2023) while, in Finland, the percentage was 3.8% (based on 2022 data). For Iceland and Sweden there was no data available. Thus, Finland seems to have the highest lifetime prevalence of LSD use in the Nordic countries (EUDA 2025; see also Karjalainen et al. 2023), although missing data from Iceland and Sweden and other methodological challenges in relation to illegal activity like drug use make these comparisons difficult.

Johnstad (2021) argues that it is difficult to generalize the consequences of psychedelics use and the user population. Some studies have obtained evidence of positive consequences and others of negative consequences, but there have not been many attempts to understand these disparate findings in a broader context and the consequences of one pattern of use are quite different from those of another pattern. When studying psychedelic naive Swedish participants attending a retreat abroad, Dunell (2024) pointed out that the participants consistently deny playful or pleasurable aspects of their intentions. Framing psychedelic use as a form of self-improvement and distancing their use from other illegal drug use is a strategy to legitimate the use of psychedelics. Articulating psilocybin use as legitimate transgression is thus aligned with dominating ideals of self-improvement. The findings of Dunnell (2024) reflect previous studies showing that psychedelic users draw on distinct discourses related to therapy, spirituality, science, performance and recreation, where the latter is the most contested (Holm et al., 2023). Research has shown that psychedelics users have many different approaches to their use and differences in usage patterns explain much of the difference in consequences of use. We know that some people take psychedelics infrequently in carefully planned sessions for spiritual, therapeutic and developmental reasons (Johnstad, 2018), while others use psychedelics very frequently for entertainment or escapist purposes (Johnstad 2021). The pleasure and euphoria experienced when using psychedelics is often overlooked in research focusing on the therapeutic effects of psychedelics (Bøhling 2017). While the use of psychedelics can induce feelings of awe (Hendricks 2018), in research contexts, this is usually linked to mystical-type experiences (Barrett & Griffiths 2018) rather than sating the human needs of curiosity and yearning for new, peak and enjoyable experiences.

Societal changes connected to drug use are often under scrutiny and there are political debates on how to tackle the increasing drug use across Nordic countries (Tham 2021). One could ask, should we even be concerned about the increasing use of psychedelics as their use on a population level seems to be associated with positive outcomes related to decreased psychological stress (Hendricks et al. 2015) and decreased opioid dependence (Jones et al. 2022) and positive health behaviors and physical health (Simonsson et al. 2022). Healthy volunteers in controlled clinical studies have also reported that their experience has been meaningful and providing insights (Elsey 2017). Still, long-term psychologically difficult experiences are also reported (Evans et al. 2023) and emergency department visits in Canada related to psychedelic use was associated with increased risk for developing schizophrenia spectrum disorder (Myran et al. 2025). Thus, these population level associations need to be complemented with better understanding of individual motivations, values and justifications for psychedelic and other drug use to comprehend the lived experiences of benefits and harms.

Ritter (2021) has argued that values necessarily shape much of the deliberation around drug policy, referring to the sense of beliefs about standards and what is important in life. While the research and use of psychedelics in various settings is on the rise, they are considered illicit drugs in the Nordics. The current legislation, various settings, motivations, understandings, research on the subject, different cultural representations and lack of a legitimate place in social reality make the use of psychedelics and values connected to their use complex phenomena to contemplate within the field of sociology. The sociology of conventions concerned with common norms and rules of action has shown that, in disputes, people tend to justify their arguments using a relatively stable repertoire of justifications (Boltanski & Thévenot 2006). The justification theory by Boltanski and Thévenot (1999) aspired to explore how individuals and groups act in real life in different types of conflicts, and how they justify their actions and views. They also outlined a general framework for analyzing the process of disputing in a complex society (Boltanski & Thévenot, 1999). According to Wagner (1999) the theory of justification is not, and does not aim to be, a normative theory of justice. Instead, it takes on the task of showing how people in everyday life's controversial situations use justifications which have a long history in the tradition of classical political philosophy (Wagner 1999; pp. 343–344). Within the context of drug research in Nordic context, justification analysis has been used to make visible the views of various stakeholder groups in a national drug policy context (Lerkkanen & Storbjörk 2023) and, for example, specific drug policy areas, such as substance use treatment (Perälä et al., 2013).

Justification theory grants actors’ critical capacity which may be and often is unequally distributed. As a starting point, the theory opens a way to analyze many processes in current complex societies more in-depth than approaches pre-assuming human agency as definitely limited by structures they operate in or the groups they belong to (Luhtakallio & Ylä-Anttila 2023). Justification theory focuses on controversies that interrupt ordinary activities, which, according to pragmatist social theorizing, make visible the foundations of social activities, as well as public argumentation around them (Lonkila et al. 2021). Doblytė (2024) argues that these cultural frames internalized through public culture, guide individuals’ aspirations and actions. Thus, analysis of justifications facilitates a better understanding of the ties between macro-level public culture, embedded in and (re)produced through social relations and institutions, and micro-level personal culture embodied by individuals (Doblytė 2024).

From a sociological perspective, the definition of the social place of various psychoactive substances, such as psychedelics, has been accompanied by negotiation, change and flexibility over different eras. Because the status of psychedelics is currently under investigation globally, we feel that a theory-based analysis of justifications is timely and provides additional understanding and insight of this complex phenomenon.

The present study aimed to determine what type of justifications does this group of Finnish participants use for their use of psychedelics, and can we interpret the place of psychedelics in Finnish society through these justifications?

## Methods

This research is part of Tsupari's PhD project. The data consist of 40 individual thematic interviews with Finnish individuals who use or have used psychedelics. The interviews were conducted by Tsupari between March and July 2022. Due to the sensitive nature of the research, we sought an ethical assessment statement from the ethical board of the University of Helsinki. Given the illegality of psychedelics and the need for confidentiality regarding the material, we have used broad subcategories for background information. The interviews were conducted with respect for the privacy of the interviewees. At the beginning of each interview, the research questions and the structure of the interview were reviewed, and participants could withdraw from the interview or decline to respond at any time.

The interviewees were recruited through the Association for Finnish Psychedelic Education & Culture, a third-sector organization whose website states that its purpose is to “promote a responsible attitude towards psychedelics by both individuals and society” (sivistysliitto.fi). Participants were recruited through a social media post and through snowball sampling during the data collection. This recruitment method ostensibly influences the profile of the people participating in the study and their justifications of the use of psychedelics. They all had broad and in-depth knowledge about psychedelics and an articulated understanding of the set and setting (Hartogsohn 2020) they considered in their own use of them.

Twenty-seven interviews were conducted in person, and the rest were online. Most participants were from the South Uusimaa region, which includes the Helsinki metropolitan area. Among the participants, 31 were men, eight were women and one participant did not want to specify their gender. The dominance of male participants is perhaps not surprising because drug use in Finland is more common among men (Karjalainen et al. 2023). It might also be that men were more interested in participating as it is more socially acceptable for men to discuss their drug use and have social circles connected to the access of drugs and psychedelics (Darcy 2020). International studies have found a stronger stigma attached to women's use of drugs and psychedelics, which may explain the lower number of female participants in this study (Viña 2025). The interviewees’ responses were most influenced by personal experiences with psychedelics and the help they sought or received from the use.

Thirty-five of the participants reported that they were currently employed, three were students and the rest did not specify their life situation. Together, the participants represented a high educational level; 30 out of the 40 participants (75%) had a university degree or a degree from a university of applied sciences. Around half of the Finnish general adult population has this level of education. The participants were of different ages: 15 were aged between 35 and 44 years, 12 were between 25 and 34 years, and 12 were between 45 and 69 years. They represented three types of household arrangements: 14 lived with a spouse, 12 in a family constellation and 13 in single-person households. This corresponds quite well to the household model division in Finnish society. Thirty-seven respondents stated that they were still active users of psychedelics, two said they had stopped and one said they were not using psychedelics but were likely to return to them at some point. The participants were not part of any explicit collective subculture, they reported voting in elections, and most considered themselves and appeared to be very regular, decent people. We have written on the values of use and experienced positive effects in another article (Tsupari et al. 2025). The participants used psychedelics one to six times a year (Tsupari 2024) and the main reasons for the use of psychedelics were self-medication, self-development and beneficial aspects, effects and psychedelic experiences. These quite ordinary but rather knowledgeable users had the means to navigate the various aspects connected to the set and setting and ways of avoiding risks connected to the use, such as mitigating dosage and stigma connected to the use of psychedelics. These aspects influence various justifications that the interviewees used and are difficult to evaluate directly.

The interviews proceeded as naturally flowing conversations, which were based on a thematic interview protocol consisting of five main themes: (1) first use experience with psychedelics; (2) drug, set and setting (i.e., meaning-making related to use context); (3) wellbeing and the significance of the use of psychedelics; (4) psychedelics in Finland; and (5) drugs and psychedelics in a societal framing. The interviews varied in length from between 40 min to 1 h and 40 min. They were transcribed verbatim and coded using Atlas.ti software by Tsupari. Through thematic analysis 15 subcategories were found from the material: (1) the interest in psychedelics; (2) perceived effect of psychedelic use on wellbeing; (3) self-medication with psychedelics; (4) psychedelic sub-culture in Finland and globally; (5) drug (the psychedelics used); (6) set; (7) setting; 8) frequency of use; (9) rationale of the use of psychedelics; (10) meaning making connected to psychedelic use; (11) motives connected to the use; (12) negative aspects connected to the use of psychedelics; (13) narratives of use and tripping; (14) technologies connected to the use of psychedelics; and (15) societal and policy conversation connected to psychedelics and drug legislation and policy.

For this study, we use the justification theory by Boltanski and Thévenot (2006) as an analytical framework to understand the rationale and justifications of people who use psychedelics. The justification framework consists of seven worlds of justification, each one having its own criteria of measuring the worth of things, people and courses of action. These worlds of justifications for arguments are:

1.World of inspiration: The emphasis is put on inspiration, uniqueness, passion, creativity, originality and emotions.
2. Domestic world: The emphasis is on traditions, personal relationships and positions in established hierarchies.
3. World of fame: The emphasis on the opinion of others, popularity, and on the opinion of celebrities and thought leaders.
4. Civic world: The emphasis is on collective, or greater good, solidarity, civil rights and democracy.
5. Market world: Money makes the world go round. The emphasis is put on goods, transactions, business and value.
6. Industrial world: The emphasis is on productivity, efficiency and solutions of science and technology. Problems are to be solved with the help of calculations, technology and science.
7. World of ecology: The ecological world is measured by the wellbeing of the natural environment.

Through a data-oriented approach, we have formed one additional world of justification, which we call the world of self. In the spirit of Boltanski and Thévenot (2006), the ideals of individualism have a strong standing in contemporary western societies. They emphasize individual's freedom, interests, rights, needs and beliefs against the predominance of other institutions in regulating the individual's behavior (Zinn 2007) and ideas of Max Weber (1922), who stated that social phenomena must be explained by showing how they result from individual actions. The focus on self includes various elements of Foucauldian concept of “care of the self” which is a major paradigm referring to lifelong work on one's body, mind and soul (Foucault, 1988). The culture of individualism is a widespread paradigm in the west and Nordic societies, and, although these factors are clearly identifiable themes in everyday life, personal decision-making and even on political spheres through the notions of neo-liberalism and individual responsibility, they are not included in the existing justification framework. The current justification framework does not include more self-driven, experience-based and pleasure-oriented actions and activities behind various human behavior.

In the world of self, the justifications of the use of psychedelics are connected to individualism through self-development, personal experiences, pleasure, curiosity and intellectual stimuli. While the renewed scientific interest in psychedelics is quite broad, certain aspects of these controversial substances are systematically overlooked, such as pleasure connected to the use of psychedelics. We have categorized the mentions connected to vivid experiences, personal insights connected to self, feelings of euphoria and pleasure, and motivation connected to curiosity to this world of justifications.

For this study, we focus on the “meanings connected to psychedelics” and the subcategories of” interest in psychedelics”, “rationale of the use of psychedelics”, “meaning making connected to the psychedelic use” and “motives connected to the use” which rose from the initial thematic analysis. The analysis is guided by a justification analysis framework (Ylä-Anttila & Luhtakallio 2016). During the interview process, it was clear that the participants used various justifications and had a reasoning behind their use of psychedelics and the analysis is based on the empirical observations during the interview process. The material was initially coded using a data-driven approach, grounded in inductive thematic analysis. This allowed patterns and themes to emerge organically from the data without imposing a predefined theoretical lens. As coding progressed, recurring patterns in how participants explained, justified or defended their psychedelic use began to surface. These patterns aligned closely with the core tenets of justification theory, particularly in how individuals construct legitimacy in the face of social norms or perceived stigma. This theoretical resonance prompted a shift toward a more theory-informed analysis, where justification theory provided a robust framework to interpret and organize the emerging themes and to gain a deeper understanding of the phenomenon in question.

To identify the worlds of justification, we have categorized systematically the participants’ responses from the mentioned subcategories into the seven original worlds of justifications, and the world of justifications which we call the world of self. This theoretical analysis includes interpretation about which world of justification the interviewees answer belongs to yet strives to remain faithful to the participants’ answers. The analysis of justifications gives us an alternative perspective on how and why the use of psychedelics might be increasing across the Nordic countries and in Finland in particular. Direct quotations from the material have been translated from Finnish into English. Unnecessary filler words and repetition have been removed.

In addition of supplementing the framework with the world of self, we have adapted the existing justification framework by categorizing the justifications connected to mystical, spiritual and religious experiences into the world of inspiration, mentions of traditional use of psychedelics into the domestic world, justifications connected to research and medical use of psychedelics and their effects on productivity, or work related inspiration, into industrial world, and mentions of strengthening the connection with nature and justifications sparking from psychedelics being naturally occurring substances to the world of ecology.

## Results

The participants tended to make a distinction between the use of psychedelics and other drugs or intoxicating substances. This was reasoned through the self-experienced therapeutic and beneficial effects of psychedelic use, such as their effect on depression and anxiety, and, in some cases, by their effect on lessening or quitting the use of cannabis or alcohol. These are common effects when psychedelics are used naturistically (Garcia-Romeau et al. 2019, 2020; Nutt 2023) and have an effect on the conceptualizations and justification of the use of psychedelics. From the original worlds of justification, only the market world was not explicitly used for justification of psychedelic use. The participants tended to use various justifications and different reasonings for their use of psychedelics and various justifications were combined logically and creatively. Their use of specific justifications was not connected to the background socio-economic factors, but was based on personal experiences, as well as ways of contemplating psychedelic experiences and their effects on everyday life.

**Figure 1.**
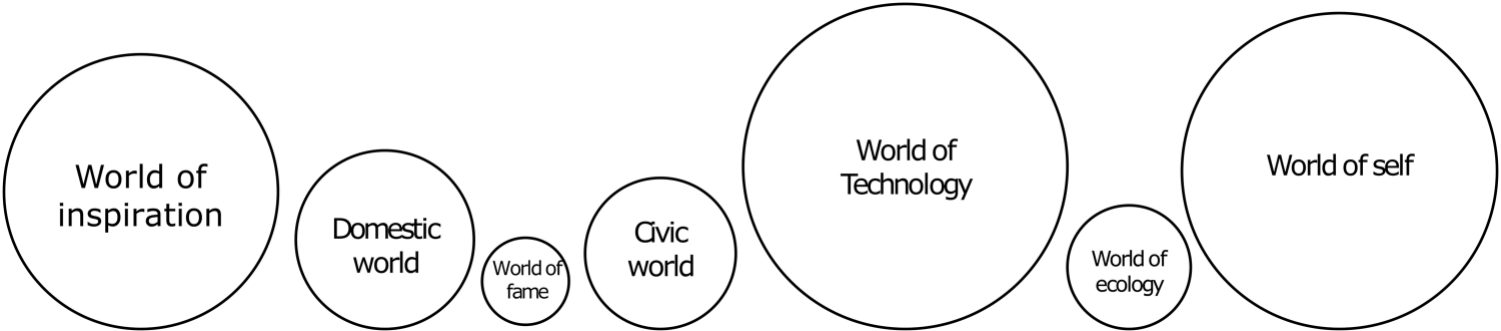
Worlds of justification for the use of psychedelics.

We have categorized justification as the main world of justification through data-oriented analysis and summarized the categorial analysis as indicated in Figure 1. The size of the shapes represented the frequency on which certain justification was used in the material. We present our findings on different justifications in the order shown in Figure 1.

## World of Inspiration

Within this framework psychedelics had focused and clarified the participants’ values, awakened blissful feelings of beauty connected to mystical, spiritual or religious experiences. These were described as “seeing behind the veil of reality”, feelings of connectedness, “all is one”-type of experiences and experiencing sanctity, if the person considered oneself religious. These conceptualizations are similar in the ways how people describe mystical experiences of which previous research has found to be important for potential benefits of psychedelic use (Kangaslampi 2023). The inspiration aspect was also described by how the psychedelic experience had affected the participants’ way of perceiving the world, their way of working as trailblazers, expansion of consciousness and the world of experience, as an ability to see the world differently, and a clear mention as a source of inspiration for voluntary artistic activities.

They work as a source of inspiration. A way to open such compartments in your mind, which might otherwise be inaccessible. They are a way to broaden your own worldview. Something like that. (man 35–44 years)

Within this world the mystical and spiritual effects of psychedelics served as the main motivation and reasoning for the use of psychedelics. Mystical, spiritual and religious experiences also morphed the justification of psychedelic use. These effects were described as powerful and altering the perception of the interviewee. The experiences under the influence of psychedelics also served as inspiration and main motivation for the use of psychedelics in cases where the use was accompanied by an element of spirituality.

Things appear in a new way. It's like an experience of freedom, the experience of holiness. The experience of connection and, also many times, fun experience. (man 45–69 years)

Artistic inspiration and spirituality are not easily acquired in everyday life, even among people who are artists or consider themselves spiritual. In these situations, the altered states of consciousness caused by psychedelics made these things more accessible, especially in the cases when the use was driven by spiritual endeavors. The felt spirituality and vivid visions under the effects of psychedelics made the participants’ beliefs and visions for artistic work “more real” and thus strengthened the involvement in these activities and their faith in specific dogma or way of life. For these interviewees, creative inspiration and spiritual matters were important enough to justify the use of psychedelics, which, in these cases, were described as entheogens, sacraments, spiritual practice or medicine, rather than illicit drugs. Similar accounts can be found in an edited volume published in 2024 by the Association for Finnish Psychedelic Education & Culture (Arkko et al. 2024) in which over 60 Finnish artists describe the effects of various psychedelics on their creativity and artistic work.

## Domestic World

Justifications connected to the domestic world, which consists of personal relationships, tradition and hierarchy might sound counterintuitive when discussing the use of psychedelics, yet these justifications were quite common. The positive use experiences and self-medication stories from trusted friends and acquaintances were described as a reason for experimenting with psychedelics. These justifications were also connected to strengthening bonds between friends, significant others and acquaintances when psychedelics were used together. This practice was described as bonding with others, psychedelic use as an activity of an inner circle of a friend group, experiencing closeness towards important people, and closeness and feelings of love towards spouse, significant other or family. If the participants’ spouse or significant other tolerated the use of psychedelics or also used psychedelics themselves, the experience highlighted the emotional, physical and intimate closeness between the couple. In cases where the significant other was not using psychedelics or was skeptical, or afraid of legal consequences, the use of psychedelics did not produce these positive effects but was rather a reason for conflicts.

The domestic world also included the justifications where the use of psychedelics was described as a part of participants’ way of life, or when the indigenous traditions and the healing rituals connected to the use of psychedelics were behind the use of psychedelics. In these cases, psychedelics were seen as a part of old cultural traditions and as an authentic way to use these substances; for example, in an organized retreat. An example of reasoning and experienced benefits is provided by from a male participant, aged 25–34 years, who used psychedelics in an organized retreat:

(after the retreat) you don’t lose your nerves so easily. In general, everything goes better, and easier, physical training goes better, food tastes better, you love your girlfriend even more… Everything falls into place, but it doesn't last forever. It lasts a week, or two, maybe three, and then it slowly fades away, unfortunately. But still, you get something out of the experience. (man 25–34 years)

Within the domestic world the justifications connected to closer family ties and better friendships are perhaps more easily understandable through the eyes of laypeople. The conflicts in close relations are generally draining and repairing difficult social relations usually improves the wellbeing of everyone involved, even when this repairment is done through illicit substances, such as psychedelics. This type of strengthening of relations could turn into problematic one if it was a regular practice, but these interviewees had usually used psychedelics once or just a couple of times to solve these issues. When psychedelics were used as an activity of the inner friend group it was seen as a group of friends’ “own thing” and had a hint of exclusivity and a way to distinct the inner group from other social circles. Yet, the use of psychedelics did not in most cases define social relations. The justifications connected to traditional use of psychedelics may seem controversial in the Nordic context, even though they are one of the driving arguments in various subcultures of psychedelic use.

## World of Fame

In the world of fame, the participants usually mentioned one or more notable people or phenomena connected to psychedelics. These justifications were built from the thoughts and ideas of the first wave psychedelic researchers’ for example, Timothy Leary, Aldous Huxley and his book “*Doors of Perception*”, Stanislav Groff, William Richards, James Fadiman and his book “*Psychedelics Explorer's Guide*”, writings of Carlos Castaneda, and the ideas of Terrance McKenna. More contemporary figures were rarely mentioned; for example, podcast host Joe Rogan and neuroscientist Sam Harris. From Finnish figures, Markku Siivola was mentioned, as well as the book by Sam Inkinen: *Tekno* (*Techno*). In this category, some of the justifications included the opinions of mystics and poets and their thoughts and ideas on the fabric of reality and esotery, history of the Grateful Dead, and the descriptions of psychedelic effects in media; for example, in the movie *Fear and Loathing in Las Vegas*. These famous figures and art styles affected the use and the way participants described their psychedelic experiences.

For maybe half a year I took these (psychedelic) truffles once a month. And then I read the works by Huxley, *Doors of Perception*, *Heaven and Hell*, and Leary's antics. At that stage I didn't have anything like a scientific point of view on it, or I didn't use them then for my own well-being, but I was a little interested in the experience. (man 35–44 years)

Of all worlds of justifications these mentions included the most humorous and anecdotal answers which resonated especially well with the most gonzoesque elements of psychedelic subculture. The notable figures of new and old were seen as experts, who had credible personal experiences or “hidden knowledge” on the subject and this made the use of psychedelics more justifiable. The countercultural movement of the 1960s seems to influence the justifications of the use of psychedelics through a wide variety of art, famous pioneers and different subcultures.

## Civic World

In the civic world, justifications were built up from greater good and the benefits of psychedelics for the individual, and society at large. These effects were described as an experience of the right to exist under the influence of psychedelics, feelings of shared humanity, strengthening of one's skill for empathy, enrichment of people's worlds of experience, better understanding of oneself and the world, heightening of compassion, connection to others through grief, gratitude, positive way of looking at humanity, breaking away from entrenched thoughts, calmness, and personal growth. These descriptions are common effects of psychedelics, which previous clinical research has reported as being part of the therapeutic effects of psychedelics (Griffiths et al. 2006, Nutt 2023). Justification for the social potential of psychedelics was usually backed by promotion of new research results and their net benefits for the whole society.

In the civic world, the potential therapeutic effects of psychedelics were emphasized as an opportunity for improved mental health for their effect on depression, trauma and anxiety, and increasing well-being for others. Treating mental health issues was seen as paramount because they are a rampant problem on a personal and societal level. In the civic world, suffering caused by mental illnesses was also seen as an injustice and source of inequality. For some interviewees, the use of psychedelics had changed their patterns of behavior. These interviewees started attending more in collective and communal activities, volunteering work and other third sector association activities. Within the civic world, psychedelics had a role for betterment of self to be better for others, and they were seen as a net positive for society through mental health treatment and betterment of individuals.

I’ve gained or received some kind of gratitude from the use of psychedelics, and a positive outlook on humanity in general. (man 25–34 years)

Within the Nordic context, the justifications of greater good, working for the betterment of society and helping the less fortunate are perhaps more prevalent than in other societies because of the history of welfare states. In addition to participating in third sector activities the greater good ethos influenced how psilocybin was acquired and distributed. In a few cases, psilocybin was purchased for a meagre fee but, in most cases, they were given or gained for free from friends and acquaintances. The belief on the net positive and “good for the cause” or the healing benefits of the drug is a clear distinction from regular market logics of illicit drug trade.

## Industrial World

The industrial world was the most common justification framework in the interviewees’ responses and had various ways of justifying the use of psychedelics. Within this framework, scientific and medical expertise in combination with the promising research results were the most common and key driving force behind these justifications. This was highlighted, for example, by describing psychedelics increasing the plasticity of the brain (Calder & Hasler 2023), as well as their benefits in the light of research for depression, anxiety, trauma and addiction. These justifications included the promotion of research and notions of psychedelics working as an accelerator on the path of psychological healing, therapy tool, emphasizing professionalism in psychedelic therapy and in indigenous traditions of psychedelics, highlighting their wasted potential in recreational use, and stating that psychedelics possess no risk of addiction.

Some participants used justifications which connected the use of psychedelics on productivity in work life or through interest in psychedelics for work related issues. For example, psychedelic landscapes worked as an inspiration for art related work, or their use was justified by their potential for mental health services in cases when the interviewee was working in the field, and aspirations for psychedelic-assisted psychotherapy. Within this world of justifications, psychedelics were seen as technologies (Hupli, 2022; Barber et al. 2023) or pharmacological intervention (Tsupari et al., 2025) which can be utilized for various purposes with the right expertise, dosage and navigation of set and setting. Seeing psychedelics as technologies is reported also by contemporary neuroscientists and clinicians working with psychedelics (Barber et al. 2023) and this framework of psychedelics as tools has emerged also among psychedelic humanities (Davis 2023). The use of psychedelics in therapeutic settings and research connected to them seems to be a major factor in the way people who use psychedelics also justify their use of these substances.

We should open our eyes to research, to investigate the potential that psychedelics have and how they can be used for benefits especially for people with depression. My experience is that even if a person doesn’t have any depression right now, it might do something good for them and possibly change their life. That psychedelics could be used for such help, legally. (man 45–69 years)

It should be noted that various therapeutic effects of psychedelics fit and can be found from other worlds of justifications, and the therapeutic effects are achievable outside research and therapy settings (Elsey 2017, Soares et al. 2022). While underlining medical, technological and science-based understanding may seem rather technocratic on a first glance, promoting the preliminary research findings and expert knowledge can be seen as a way to tackle the stigma connected to use of psychedelics in Finland, where they are classified as illicit substances This is achieved by defining psychedelics as legitimate medicines and therapy tools within certain settings, not as illicit drugs.

## World of Ecology

Within the world of ecology, psychedelic experiences strengthened the importance of nature and ecology through the effects of psychedelics. These experiences were described as “all is one”-mystical type experiences, the strengthening of nature experience and nature connections and appreciating the beauty of nature, finding more appreciation for all living things, and moving towards a more ecologically sustainable lifestyle. In the world of ecology, natural environments functioned also as a stage or setting for psychedelic experiences, and these psychedelic experiences in nature influenced the ways interviewees saw themselves as part of the natural order.

This is a very general realization that “we are all one” and this kind of thing. And that you can really form a spiritual relationship with some tree or flowers. I had this psychedelic experience on mushrooms once. That I dived to lie down among the violets and then they breathed around me, it was a really holistic experience. (man 25–34 years)

These findings connected to the world of ecology are like the findings by Forstmann & Sagioglou (2017) who have pointed out empirical connection from experiencing unity and connection with one's environment to empathy-related phenomena and pro-environmental behavior. Their research highlighted that the perception of being part of the natural world, rather than being separate from it, is largely responsible for the increased pro-environmental behavior that people report in relation to psychedelic use (see also Gandy et al. 2020). For participants who used only plant-based psychedelics, nature was also seen as a provider for these substances. In these cases, psychedelics were seen as a “gift from nature” and their natural occurrence was seen as a part of justification for their use.

Within this world of justifications, the use of psychedelics seemed to answer the yearning for a more natural and a closer-to-nature way of life, including in relation onés diet. The notion of changing one's eating habits can reduce the carbon footprint of the person in hand but also possess some benefits for mental and physical wellbeing.

## World of Self

While the original justification theory does not include the world of self, the participant's answers and our interpretation inspired us to add new theoretical elements to the original theory. In this world, the justifications are connected to individualism, self-development and personal and new experiences, pleasure, and intellectual stimuli. We are highlighting these justifications because pleasurable aspects are often overlooked, especially in contemporary research connected to psychedelics and drug use research in general (Dennis & Farrugia 2017). While the main motivation for these participants was on the benefits and positive effects of psychedelics, their reasoning and justifications also included some recreational elements. Bøhling (2017) has argued that pleasure is an important and understudied aspect of psychedelic experiences and practices. The use of psychedelics was in many cases sparked by curiosity, craving for new types of experiences, wishes to experience altered states of consciousness, sensory changes and something that might seem hidden or mysterious in everyday life. The effects of psychedelics were described as feeling good, happy or euphoric during or after the use, experiencing feelings of love and other strong emotions and meaningful insights, seeing beautiful or geometrical hallucinations, visuals and other types of aesthetic experiences, and altered senses or strengthening of senses, such as merging with music. These are typical effects psychedelics produce when consumed also in clinical settings (Andersen et al. 2021). Personal effects were described by having meaningful and peak experiences, release of emotional stress, increase of openness and handling of difficult emotions. In a few cases, it was clearly stated that psychedelics had more recreational and entertainment aspects in certain settings. These moments contained laughter and were described as relaxation and having fun, defining the use of psychedelics as a hobby or as a way of spending time, celebrating or breaking away from everyday life, or as a means of intensifying concerts and other art experiences. For these interviewees, the pleasurable and sensory aspects of psychedelics were an important part of the experience, even if the main motivation was on self-improvement, self-medication or connected to spiritual or religious ventures as we can interpret from the next sample:

I use them in the sense of healing. I have to say that sometimes I enjoy the trips. There are some fantastic aspects in them, but that's not the point. Spiritual growth is what I'm looking for, not recreational use. But then San Pedro, for example, is taken during the day in a group and the effect is more social. So, if I may use the saying, it's pretty damn fun. But the deeper essence is the most important. (man 45–65 years)

In the world of self, psychedelics were in some cases used as developmental tools on the project of self. While the pleasure and experience-based justifications were dominating in this world of justification, there were numerous mentions of how one has a responsibility to take care of oneself and aspire to become a better version of oneself. These reasonings were often entwined and seen as different positive aspects of psychedelic use, rather than seeing pleasure, curiosity and vivid experiences as separate or purely negative, and self-development and self-medication as purely positive.

Psychedelics are a rare treat for me. I can see that there's an entertainment aspect, even partly in my own use. I always try to make sure that the setting is safe to minimize the risks. At the same time, they work as a kind of tool for working on yourself, that you see new sides and new perspectives and experience new thoughts and everything. After the experiences I try to hold on to the positive feelings and effects in everyday life. And I find it really meaningful. (woman 18–24 years)

Perhaps the most surprising part of this world of justification was on how open the interviewees were about their enjoyment of the psychedelic effects, even in cases when they were used for self-medication. These conversations included mentions of fun and enjoyment, awe and even celebrating the effect of psychedelics boosting senses in more recreational settings. This is an aspect which is quite often downplayed within the new wave of psychedelic research, when, in fact, some people enjoy the psychedelic effects, and some will probably do so even in legitimate research and therapeutic settings. This challenges our notions of seeing medical treatment, self-development and curing mental illnesses as gruesome, tiring and even painful processes when, in fact, it might contain all the negative aspects, but also have a spark of enjoyment, feelings of gratefulness and awe.

## Discussion

In the present study, we analyzed the motives and justifications of psychedelic use of 40 Finnish interviewees using Boltanski and Thévenot's (2006) justification analysis framework. When discussing delicate and controversial topics, such as the use of psychedelics, it is not surprising that the motivations and justifications are blurred and entwined. The justifications varied from personal reasons to societal aspirations and from spiritual to technological justifications. These varied justifications show us that currently psychedelics do not have a clear cultural place in the Finnish context. In public spheres and legislation, they are seen as illicit drugs, but, in user narratives, the use of psychedelics is seen through various benefits, personal experiences and by distinguishing the use of psychedelics from other illicit drugs. In addition, the interviewees used various justifications and even combined justifications from different worlds fluently. This was especially the case where psychedelics were used as a self-help remedy for various mental conditions. In these cases, the use of psychedelics could have highlights and justfications from various worlds, such as having elements from the world of industry when psychedelics worked as a remedy for mental health issues, yet the interviewee would at the same time describe the psychedelic experience as intriguing, vivid or euphoric. This is the interpretive aspect of our analysis, although we believe we have stayed true to our participants’ expressions and meanings.

While the justifications explicitly connected to the market world were not found in the interview material, these justifications are connected to the global new wave of psychedelic research through the interest of pharmaceutical companies and venture capital (Yaden et al. 2022). This branch of research on psychedelics is connected to certain neoliberal economic contexts driven by processes such as medicalization, legalization, technophilia and optimism, as well as neocolonialism (Noorani 2020; Hauskeller & Schwarz 2023), which in turn affect justifications around their use. Thus, while the market world was not present in our interviewees’ justifications, this could change if psychedelic medicine becomes more established in the Finnish or Nordic context.

The justifications in the world of inspiration resonated with those interviewees, who are perhaps more spiritual. This world of justification is strongly linked to the mystical experiences which the use of psychedelics might cause in certain settings and with high enough doses. Research has found these experiences important and, in some cases, positively life-altering (Barrett & Griffiths 2018, Andersen et al. 2021).

The justifications in the world of industry show that the new wave of psychedelic research has a major impact on the lived experiences of people who use these outside research and therapy settings. The participants used the terminology and the new research findings fluently in their justifications. This is most likely due to personal experiences with self-medication and the positive effects that the use of psychedelics had for these interviewees. Justifications connected to the medical and scientific expertise and self-medication are perhaps easier to accept among people who have not used psychedelics because all Nordic countries are post-industrial, post-modern welfare states.

Justifications in the world of self were connected to personal experiences, such as experiencing powerful or euphoric emotions and states, seeing vivid hallucinations and fractals, gaining personal insights for self-development, and relaxation and spending time in more recreational ways in altered states of consciousness. The justifications connected to freedom, interests, rights, needs or beliefs are not a surprising finding because these values are seen as essential in the western world. On the one hand, the use of psychedelics can be seen as a personal effort of tackling mental health challenges or as a tool for self-improvement (Soares et al. 2022; Tsupari et al. 2025). On the other hand, their use has aspects which can be seen as recreational, motivated by a desire for new experiences or a yearning for an altered way of being. We are not trying to downplay the reported benefits or discredit the lived experiences of the participants by pointing out that their beneficial use of psychedelics has also an experience and pleasure-driven aspect. We are highlighting this because these aspects are overlooked or downplayed even in the participants’ reports of experiences in the Nordics (Dunell 2024).

The new wave of psychedelic research often disregards the fact that some people find the psychedelic experience interesting, euphoric or even fun, even in cases where the use is therapeutic, beneficial or has spiritual aspects. Goldy et al. (2024) have proposed that understanding the role of positive emotions within the context of psychedelic experiences could help elucidate the connection between psychedelics’ acute subjective effects and therapeutic outcomes. The lived experiences of naturalistic psychedelic users show us that pleasure and euphoria are essential parts of psychedelic experience and could have an important role also in the therapeutic use of psychedelics. These aspects should be considered more in medical and therapeutic research of psychedelics.

## Limitations

The justification framework by Boltanski & Thévenot (2006) was designed for analyzing public and work-related discourses. Although our data comprises private interview material, people tended to use the similar justifications as in public debates. Our interviewees were predominantly male, urban, well-educated and employed. This probably influences the used justifications and the findings of the research. The existing justification framework does not include personal experiences and individualism in any world, yet people *de facto* use these as arguments and justifications for actions in public and private spheres. This offers new avenues for developing the justification analysis, especially in the contemporary societies of individualism and social media.

Due to ethical reasons and the fact that use of psychedelics is illegal in Finland, we did not save the contact information of the participants; thus, we were unable to obtain feedback on our interpretations. This was another limitation of the study, yet this offers another avenue for future research where co-production of knowledge is used as a method.

## Conclusions

To our knowledge, this is the first study to use the justification framework for analyzing reports of psychedelic use in the Finnish context. All worlds of justification appeared in the data material, except for the market world. We included an additional world of self to capture justifications connected to various personal experiences, including pleasure which is often overlooked in modern clinical research.

Analyzing justifications used by Finnish interviewees illuminate not only their personal experiences with psychedelics, but also the broader cultural narratives the interviewees draw upon. Various subcultures connected to psychedelics, media presentations and the new wave of psychedelic research affect the way people justify the use of these currently highly debated substances. Highlighting the positive effects of psychedelics and drawing legitimacy through research results and medical expertise can be seen as a way of dealing with stigma connected to the use of psychedelics and as a strategy of redefining the use of psychedelics as self-medication, spiritual practice or as an innovative solution for mental health issues under prohibitionist drug policy. The new wave of psychedelic research and personal experiences of the interviewees are a direct contrast to Finland's current drug policy, where all illicit drugs are seen as a monolithic category which causes crime, death and despair. The new wave of psychedelic research offers alternative narratives to frame the use of psychedelics and know-how on how, which psychedelics and where they should be consumed. This trend is most likely behind at least part of the increase in the use of psychedelics because self-medication with psychedelics may seem like a viable alternative when mental health problems are increasing in society and Finland is currently going through a rather severe austerity policy.
